# AIGO: Towards a unified framework for the Analysis and the Inter-comparison of GO functional annotations

**DOI:** 10.1186/1471-2105-12-431

**Published:** 2011-11-03

**Authors:** Michael Defoin-Platel, Matthew M Hindle, Artem Lysenko, Stephen J Powers, Dimah Z Habash, Christopher J Rawlings, Mansoor Saqi

**Affiliations:** 1 Multidisciplinary Centre for Integrative Biology, The University of Nottingham, UK; 2 Department of Biomathematics and Bioinformatics, Rothamsted Research, Harpenden, UK; 3 Department of Plant Science, Rothamsted Research, Harpenden, UK

## Abstract

**Background:**

In response to the rapid growth of available genome sequences, efforts have been made to develop automatic inference methods to functionally characterize them. Pipelines that infer functional annotation are now routinely used to produce new annotations at a genome scale and for a broad variety of species. These pipelines differ widely in their inference algorithms, confidence thresholds and data sources for reasoning. This heterogeneity makes a comparison of the relative merits of each approach extremely complex. The evaluation of the quality of the resultant annotations is also challenging given there is often no existing gold-standard against which to evaluate precision and recall.

**Results:**

In this paper, we present a pragmatic approach to the study of functional annotations. An ensemble of 12 metrics, describing various aspects of functional annotations, is defined and implemented in a unified framework, which facilitates their systematic analysis and inter-comparison. The use of this framework is demonstrated on three illustrative examples: analysing the outputs of state-of-the-art inference pipelines, comparing electronic versus manual annotation methods, and monitoring the evolution of publicly available functional annotations. The framework is part of the AIGO library (http://code.google.com/p/aigo) for the Analysis and the Inter-comparison of the products of Gene Ontology (GO) annotation pipelines. The AIGO library also provides functionalities to easily load, analyse, manipulate and compare functional annotations and also to plot and export the results of the analysis in various formats.

**Conclusions:**

This work is a step toward developing a unified framework for the systematic study of GO functional annotations. This framework has been designed so that new metrics on GO functional annotations can be added in a very straightforward way.

## Background

Given the advent of high-throughput sequencing technologies and the resulting data explosion, there is an urgent requirement to provide electronically inferred functional annotations (FAs) for proteins whose biological functions have not yet been determined by experimental investigation. This has given rise to a large number of annotation inference pipelines [1-6] which are used routinely to produce new annotations that are stored in a variety of publicly accessible databases. Therefore, when looking for FAs related to a particular organism, it is commonplace to find several resources each providing annotations that have been predicted using different pipelines, at different times and under different conditions. Consequently, end-users of FAs have to decide between multiple resources. However, since there is no single accessible and objective way to compare these FAs, these decisions are in many cases made arbitrarily, whereas different biological problems may require consideration of annotations from different points of view.

Where pipeline outputs reference a controlled vocabulary or ontology, relative comparisons of these outputs can be made by using a reference protein-set as a common input. Many pipelines endeavour to annotate gene products to the three aspects of the Gene Ontology (GO): molecular function (MF), biological process (BP), and cellular component (CC). For each GO aspect, a given gene product can therefore be annotated with a set of GO terms. GO provides not only a unified and shared controlled vocabulary for annotation but a structured representation of knowledge. The power of this annotation system over a flat controlled vocabulary is twofold. Firstly, it can be used to evaluate the functional distance between genes [7] by quantifying the difference between their annotation sets based on the semantics codified in the structure. Secondly, it defines a hierarchy between the GO terms that leads to annotations being more or less specific, *i.e. *more or less biologically informative.

The aim of this study is to define and implement an ensemble of measures that provide both a quantitative and a qualitative description of GO FAs and also allows a comprehensive inter-comparison between them. So far, a plethora of such measures have been proposed but no attempts have yet been made to define and implement a unified framework where FAs can be compared in a systematic way. A notable exception is the Blast2GO suite [8] where several metrics describing FAs are implemented together, for example to measure the specificity of annotations or to automatically compare groups of GO terms. However in Blast2GO, these metrics are only used to measure specific aspects of Blast2GO performance and furthermore, the Blast2GO suite is not designed to compare FAs issued by other pipelines. In most studies, the different properties of FAs are implemented and studied independently *e.g. *[9,10]. In addition to facilitating the comparison of annotation sets produced by various annotation pipelines, having a common unified framework that implements these metrics would assist in the evaluation of new metrics, the exploration of the influence of parameters in annotation pipelines and the monitoring of annotations over time.

In this paper, we propose a set of twelve metrics and implement them in a Python open source library named AIGO for the Analysis and the Inter-comparison of GO functional annotations. We demonstrate the use of the AIGO library for evaluating and comparing the output of annotation pipelines using three illustrative examples.

## Methods

In this section, we define a set of twelve metrics (nine for the analysis and three for the inter-comparison of FAs), each of them describing particular features of FAs, see Table 1.

**Table 1:** Features of functional annotations described by AIGO metrics

Measure	Feature of a functional annotation
Coverage	Proportion of genes annotated by a FA
Richness	Proportion of GO terms used by a FA
Nb. of annotations	Number of annotations of a given gene
Coherence	Similarity of the terms in a set of annotations
Compactness	Similarity of the annotation sets in a FA
Specificity	Specificity of the annotations for a given gene
Information Content	Informativeness of the annotations for a given gene
Redundancy	Proportion of redundant terms in a set of annotations
Obsolescence	Proportion of obsolete terms in a FA

Semantic similarity	Degree of functional relatedness between two annotation sets
Hierarchical Precision	Proportion of the annotations in a given FA being also found in a gold-standard
Hierarchical Recall	Proportion of the annotations in the gold-standard being also found in a given FA

The different metrics computed by AIGO allow describing particular features of functional annotations.

### Definitions of metrics

For any given organism Ω, we call functional annotation, noted ℱ, the mapping between a set of gene products *G*_Ω _from this organism and some functional data. In practice, a set of gene products from an organism can be defined in various ways, ranging from a comprehensive list of proteins (if this organism has been well studied) to a list of ESTs assembled into potentially incomplete or inaccurate UniGenes (if the organism has not yet been sequenced), see [11]. A common scenario is that the set of gene products corresponds to some defined reference set: for example the list of target sequences from a microarray. The functional data are usually provided as annotation sets containing terms from controlled vocabularies or reference ontologies. We note  a given ontology, defined as a direct acyclic graph connecting a set of terms $T_{O}$. In this study,  can be any of the three aspects of GO (*i.e. *BP, MF and CC). More formally, a functional annotation of a set of gene products *G*_Ω _over a given ontology , is a map ${ℱ}_{O}:G_{\Omega }\to 2^{O}$ that associates with each gene product *g *∈ *G*_Ω _a subset of $T_{O}:{ℱ}_{O}\left(g\right)\subseteq T_{O}$.

### A. Quantity of annotations

We define the breadth of *coverage *of ${ℱ}_{O}$ as the proportion of gene products from a given organism that are annotated to at least one term from the reference ontology .

$$Cov\left({ℱ}_{O}\right)=\frac{\left|GA_{O}\right|}{\left|G_{\Omega }\right|}$$

where $GA_{O}=\left\{g|g\in G_{\Omega ,}{ℱ}_{O}\left(g\right)\neq \mathrm{\emptyset ̸}\right\}$. We define the *richness *of ${ℱ}_{O}$ as the proportion of $T_{O}$, the set of terms from , which is assigned to at least one gene product from ${ℱ}_{O}$. We note:

$$Rich\left({ℱ}_{O}\right)=\frac{1}{\left|T_{O}\right|}|\left\{t|t\in T_{O},GP_{O}\left(t\right)\neq \mathrm{\emptyset ̸}\right\}|$$

where $GP_{O}\left(t\right)=\left\{g|g\in G_{\Omega ,}\;t\in {ℱ}_{O}\left(g\right)\right\}$. Given a set of annotated gene products $GA_{O}$, another statistic of interest is the *number of annotations * with which a gene product is associated with respect to the ontology . We note, $\forall g\in GA_{O}$

$$N\left({ℱ}_{O},g\right)=|{ℱ}_{O}\left(g\right)|$$

### B. Diversity of annotations

To gain a more qualitative insight into FAs, it is useful to know for each gene product, how diverse its annotations are. We propose to measure the *coherence *of annotation sets, $\forall g\in GA_{O}$

$$Coh\left({ℱ}_{O},g\right)=\frac{\sum_{i\in F_{O}\left(g,\right)}sim\left(\left\{i\right\},{ℱ}_{O}\left(g\right)-\left\{i\right\}\right)}{N\left({ℱ}_{O},g\right)},$$

with *sim*(*S*_1_,S_2_) providing a measure of the *semantic similarity *between two annotation sets *S*_1 _and *S*_2_. In a general sense, measuring the semantic similarity between two biological entities is equivalent to assessing the degree of functional relatedness between these entities by evaluating the similarity between their annotations. Further details on the semantic similarity measures implemented in AIGO are given below in the paragraph Inter-comparison of functional annotations. Likewise, to estimate the degree of heterogeneity of an entire FA, we define its *compactness *with respect to a given ontology as the mean semantic similarity between all its annotation sets, such that:

$$\begin{array}{c} Comp\left({ℱ}_{O}\right)=\frac{\sum_{g\in GA_{O}}avSim\left(g,{ℱ}_{O}\right)}{\left|GA_{O}\right|}\\ avSim\left(g,{ℱ}_{O}\right)=\frac{\sum_{g^{\prime }\in GA_{O-\left\{g\right\}}}sim\left({ℱ}_{O}\left(g\right),{ℱ}_{O}\left(g^{\prime }\right)\right)}{|GA_{O}|-1} \end{array}$$

### C. Informativeness of annotations

Not all FAs are equally informative and the amount of useful information that they provide needs to be carefully appraised. One possible way to assess the usefulness of a FA is to look at the relationship between the annotated terms in the reference ontology. In GO, the relationships between the terms has the form of a direct acyclic graph, where ancestral terms are less specific than their descendants. For a given gene product, we define the specificity of its set of annotations as the mean number of ancestors over all annotated terms, $\forall g\in GA_{O}$

$$Spec\left({ℱ}_{O},g\right)=\frac{1}{N\left({ℱ}_{O},g\right)}\sum_{i\in F_{O}\left(g\right)}|A_{O}\left(i\right)|$$

where $A_{O}\left(i\right)$ is the set containing a term *i *and all its ancestors in . We also define the *redundancy *of an annotation set as the proportion of terms in the set that are ancestors of other terms in that set, $\forall g\in GA_{O}$

$$Red\left({ℱ}_{O},g\right)=\frac{1}{N\left({ℱ}_{O},g\right)}|\left\{i|i,j\in {ℱ}_{O}\left(g\right),i\in A_{O}\left(j\right)\right\}|$$

A common criticism of GO structural metrics is that they assume a homogeneous difference in GO term specificity across all edges of the GO directed graph. Information Content (IC) based measures attempt to address this problem by providing metrics for the specificity of a term based on the frequency of its usage. Several studies have suggested that IC may provide a superior measure of semantic distance compared to graph structural metrics [12,13], but IC is sensitive to annotation bias and changes over time in non-reference organisms [14]. IC is based on Shannon's information theoretic measure [15], where:

$$IC\left(i\right)=-log_{2}p\left(i\right)$$

and probability *p*(*i*) of a term is the frequency of a term and of its ancestors in a FA.

$$p\left(i\right)=\frac{\sum_{t\in A_{O}\left(i\right)}|GP_{O}\left(t\right)|}{\sum_{j\in T_{O}}|GP_{O}\left(j\right)|}$$

As with most of the biomedical ontologies, GO is dynamic and the structure and content evolve continuously, with new versions released at regular intervals. Almost all features of GO can undergo change: the definition of terms can be revised and the relationship between terms can be deleted. It is often the case that terms are made obsolete and replacement terms are proposed. However, it is also possible that no appropriate alternatives can be found to replace an obsolete term and so it becomes *de facto *disconnected from the rest of the ontology. In this type of situation, the annotations carry virtually no useful information. We define the *obsolescence *of a functional annotation ${ℱ}_{O}$, with respect to a given ontology, as the proportion of obsolete annotations for which no alternative have been proposed. Let ${\tilde{T}}_{O}\subseteq T_{O}$ be the set of obsolete terms, then we have

$$Obs\left({ℱ}_{O}\right)=\frac{\sum_{t\in \left\{t|t\in {\tilde{T}}_{O},\;GP_{O}\left(t\right)\neq 0̸\right\}}|GP_{O}\left(t\right)|}{\sum_{t\in \left\{t|t\in T_{O},\;GP_{O}\left(t\right)\neq 0̸\right\}}|GP_{O}\left(t\right)|}$$

### D. Inter-comparison of functional annotations

It is possible to obtain a general comparison of several FAs by contrasting the values they report for the nine metrics described above. However, a deeper examination is sometimes required, for example, to know how many gene products are commonly annotated by two or more FAs, what these gene products are, and how different/similar their annotation sets are within the FAs in question. In this section, we describe three metrics for the inter-comparison of FAs.

The AIGO library will generate and display Venn diagrams to compare the coverage of FAs. When gene products are annotated by at least two FAs, AIGO can compute the *semantic similarity *between their sets of annotations. This functionality is particularly helpful for identifying highly dissimilar annotation sets and therefore has the potential to detect errors in FAs. Numerous metrics have been proposed to evaluate the semantic similarity between annotation sets. In the AIGO library, three semantic similarity metrics are implemented: (i) the Resnik metric based on the most informative common ancestors of GO terms [16], (ii) a graph-based metric known as the Czekanowski-Dice similarity (c.f. [17] to see how this can be used in the context of the Gene Ontology) and (iii) another graph-based metric called GS2 [18] which behaves similarly to the measure developed by Wang *et al. *in [19] but is more computationally efficient since it can measure the similarity of a set of genes in linear time in the size of the set. Because of its greater efficiency, the GS2 metric has been used for all the semantic similarity calculations in this study.

Finally, the last two metrics implemented in AIGO to compare FAs are the *hierarchical precision *and the *hierarchical recall *[20]. These two metrics describe the accuracy of FAs according to a reference FA, *i.e. *a gold-standard, taking into account the hierarchical nature of the Gene Ontology. The precision corresponds to the proportion of the annotations in a given FA that are also found in the gold-standard, whereas the recall corresponds to the proportion of the annotations in the gold-standard that are also found in a given FA. More formally, we note for ${ℱ}_{O}$, a functional annotation, and $G_{O}$, a gold-standard, $\forall_{g}\in \left\{g|g\in G_{\Omega ,}\;{ℱ}_{O}\left(g\right)\neq 0̸,\;G_{O}\left(g\right)\neq 0̸\right\}$

$$\begin{array}{c} hPrecision\left(g,{ℱ}_{O},G_{O}\right)=\frac{\sum_{t\in F_{O}\left(g\right)}sim\left(s,g,G_{O}\right)}{\left|{ℱ}_{O}\left(g\right)\right|}\\ sim\left(s,g,G_{O}\right)=MAX_{t\in G_{O}\left(g\right)}\frac{\left|A_{O}\left(t\right)\cap A_{O}\left(s\right)\right|}{\left|A_{O}\left(t\right)\right|}\\ hRecall\left(g,{ℱ}_{O},G_{O}\right)=\frac{\sum_{s\in G_{O}\left(g\right)}rSim\left(s,g,{ℱ}_{O}\right)}{\left|G_{O}\left(g\right)\right|}\\ sim\left(s,g,{ℱ}_{O}\right)=MAX_{t\in F_{O}\left(g\right)}\frac{\left|A_{O}\left(t\right)\cap A_{O}\left(s\right)\right|}{\left|A_{O}\left(t\right)\right|} \end{array}$$

### Implementation: the AIGO library

The different metrics presented in the previous section have been implemented in a Python library, named AIGO, for the Analysis and the Inter-comparison of GO functional annotations. AIGO is an open source library distributed under the GNU General Public License v3 and source code, documentation and illustrative examples can be found here: http://code.google.com/p/aigo. AIGO is an object oriented Python library implementing a collection of classes to load, manipulate, analyse and inter-compare FAs. The library can read annotation files in various formats such as "GO Annotation File 2.0", Affymetrix annotation files (TAF format) and generic mapping files. Additionally, AIGO can read GO files in xml OBO format and gene set reference files in different formats, including the FASTA format. AIGO can automatically create graphics showing the results of the comparison of multiple FAs, including the distribution of the metrics and draw annotated directed acyclic graphs of GO. All the results can also be exported into simple text files or into Microsoft Excel files. To assess the statistical significance of the results, AIGO also provides classes to perform permutation tests by randomizing existing FAs or by sampling FAs directly from GO.

**Figure 1:**
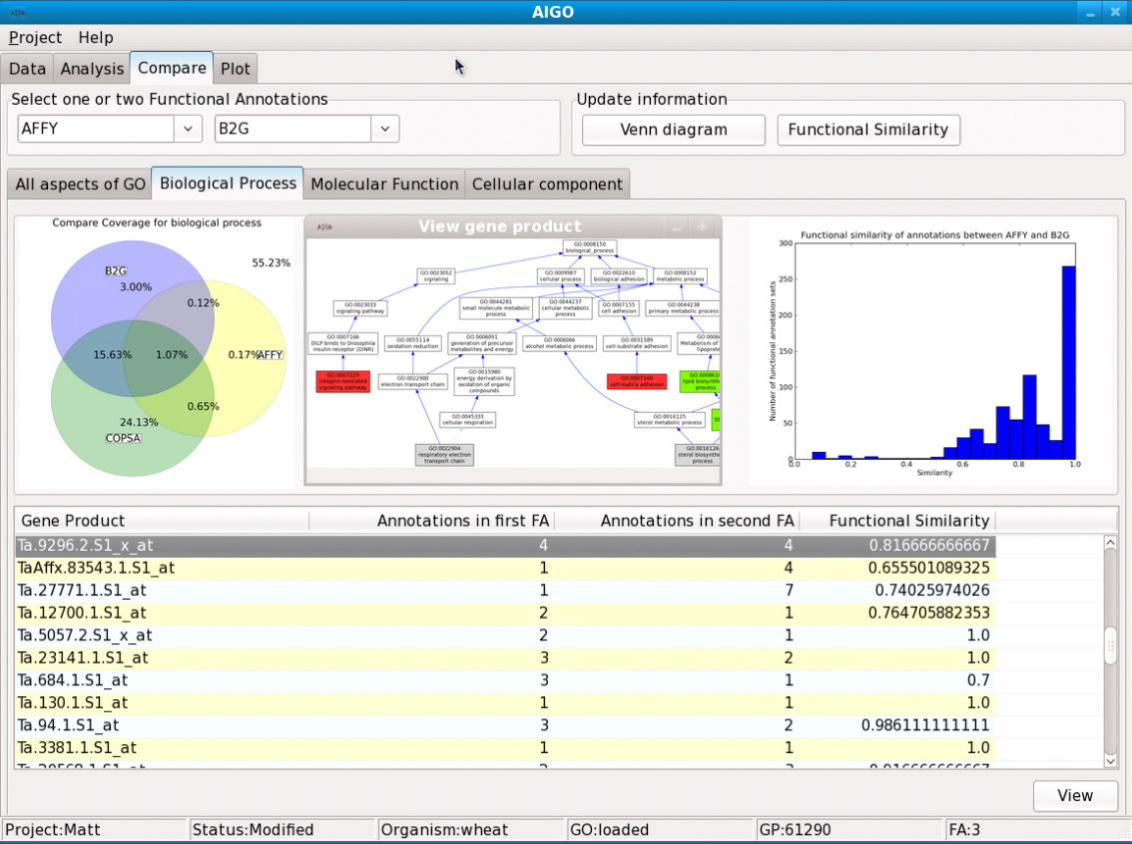
**The AIGO graphical user interface**. The AIGO graphical user interface allows loading gene ontology files, sets of reference gene products and functional GO annotations in various formats. It also allows the user to compute AIGO metrics, and then display and export the results in various formats.

A simple Graphical User Interface (see Figure 1) allows access to most of the functionalities of the library in a very simple and efficient way. It is worth noting that AIGO has been designed so that new metrics can be easily added to the library. Once registered, the new metrics automatically appear in the user interface, and the results of their computation will be automatically plotted and exported.

Most of the operations that AIGO performs are not computationally intensive and therefore do not require much CPU time or memory. For example, the first case study presented in the Results section took exactly 2 minutes to run on a dual core CPU (2.8 Ghz). In practice, the computation of most of the statistics is very fast, with the exception of the those involving semantic similarity. The GS2 metric is very scalable, since it is computable in linear time in the size of the set of gene products, however computing the *Coherence *and the *Compactness *statistics can still take several minutes. Another rather demanding operation is plotting the direct acyclic graph induced by a set of annotations: more precisely, the layout of the nodes can take one hour in the worst case.

## Results and Discussion

### Example 1: Inter-comparison of annotation pipelines

The unified framework implemented in the AIGO library was used to analyse and compare three publicly available FAs for the Affymetrix GeneChip Bovine Genome Array (see Additional file 1 - Reference of Affymetrix GeneChip genome arrays). These annotations are provided by Affymetrix (AFFY) [6] and by two cross-species annotation pipelines: Blast2GO (B2G) [4,21] and ArrayIDer (AID) [22]. The set of gene products used as a reference in this study corresponds to the 24,128 target sequences of the Affymetrix bovine array. For the three FAs mentioned above, Table 2 reports the values of nine statistics of interest computed using the AIGO library. We note that both AFFY and B2G provide a small number of obsolete terms for which no alternative exist. We chose to remove these terms from AFFY and B2G before computing the other metrics.

**Table 2:** Properties of Bovine functional annotations

Measure	AFFY	B2G	AID
	GO Biol ogical Process		
Obsolescence	0.02%	0.04%	0.0%
Coverage	41.2%	28.8%	50.1%
Richness	19.4%	24.1%	22.7%
Avg. nb annot.	3.7	5.3	4.2
Avg. Coherence	0.6	0.5	0.6
Compactness	0.3	0.3	0.4
Avg. Specificity	12.2	13.4	11.3
Avg. IC	5.4	6.0	5.1
Avg. Redundancy	18.1%	2.3%	18.4%
	GO Molecular Function		

Obsolescence	0.0%	0.0%	0.0%
Coverage	49.3%	29.8%	52.3%
Richness	22.4%	24.5%	24.9%
Avg. nb annot.	3.2	2.8	3.3
Avg. Coherence	0.6	0.5	0.6
Compactness	0.5	0.5	0.5
Avg. Specificity	5.1	5.7	5.0
Avg. IC	4.2	4.9	4.2
Avg. Redundancy	32.7%	0.3%	31.8%
	GO Cellu lar Component		

Obsolescence	0.0%	0.0%	0.0%
Coverage	42.7%	30.3%	51.5%
Richness	24.6%	31.4%	27.8%
Avg. nb annot.	2.7	2.5	2.7
Avg. Coherence	0.8	0.7	0.8
Compactness	0.6	0.6	0.6
Avg. Specificity	8.9	11.7	8.3
Avg. IC	3.0	3.8	2.8
Avg. Redundancy	33.2%	0.1%	31.9%
	All three aspects of GO		

Obsolescence	0.01%	0.02%	0.0%
Coverage	56.4%	33.3%	60.4%
Richness	20.7%	24.9%	23.8%
Avg. nb annot.	3.2	3.5	3.4
Avg. Coherence	0.7	0.6	0.7
Compactness	0.5	0.5	0.5
Avg. Specificity	8.7	10.3	8.2
Avg. IC	4.2	4.9	4.1
Avg. Redundancy	28.0%	0.9%	27.4%

The statistical properties of functional annotations in Affymetrix, Blast2GO, ArrayIDer generated by AIGO for each aspect of the gene ontology.

### Analysis of Functional Annotations

AID has the greatest breadth of *coverage*, with a majority of target sequences (60.4%) having at least one annotation from at least one of the three aspects of GO, whereas B2G systematically reports the lowest *coverage *results. Unexpectedly, despite this smaller *coverage*, B2G contains more diverse GO terms than the other FAs, with *richness *(*i.e. *the proportion of GO terms assigned to at least one sequence) almost equal to 25% (all aspects of GO). The *compactness *statistic (which is a measure of the diversity of the annotation sets) is similar across the three FAs; this indicates that their annotation sets are distributed in a similar way across the ontology.

**Figure 2:**
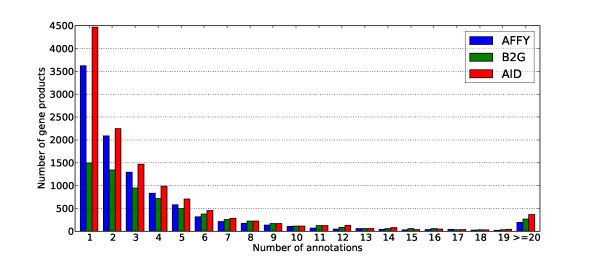
**Number of GO annotations (Biological Process) per gene in *Bos taurus***. Distributions of the size of the annotation sets for the Biological Process annotations provided by Affymetrix, Blast2go and ArrayIDer for the Affymetrix GeneChip Bovine Genome Array.

For the three FAs, the annotated gene products are associated with 3 or 4 terms on average. However, as illustrated in Figure 2 for AFFY, B2G and AID, the distributions of the size of the annotation sets are strongly positively skewed. Consequently, while a large proportion of the annotation sets contain only one term, some other annotation sets are surprisingly large. For the BP aspect of GO, the largest set consists of 96, 99 and 156 terms for AFFY, B2G and AID respectively. We note that for both AFFY and B2G, the largest set corresponds to the same probe-set: Bt.13141.1.S1_at. This probe-set is associated with the "BCL2 B-cell CLL/lymphoma 2" gene, a member of the large BCL2 family involved in the regulation of apoptosis. There is a strong possibility that slightly different annotations coming from all the members of this family have been electronically inferred and transferred to the Bt.13141.1.S1_at probe-set, which would explain the size of its annotation set (see Additional file 2 - Annotations of Bt.13141.1.S1_at).

**Figure 3:**
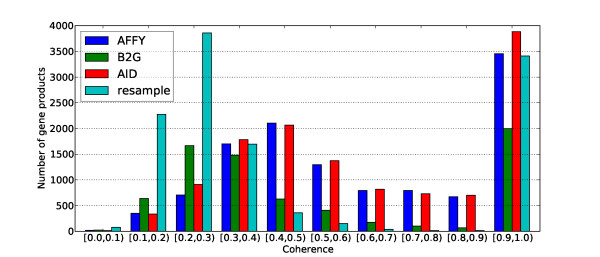
**Coherence of GO (Molecular Function) annotation sets in *Bos taurus***. Distributions of the coherence of the annotation sets for the Molecular Function annotations provided by Affymetrix, Blast2GO, ArrayIDer and a randomly sampled functional annotation where original AFFY annotations are replaced by random annotations, uniformly drawn from GO, for the Affymetrix GeneChip Bovine Genome Array.

This very high proportion of sets containing only a single term has a clear impact on the *coherence *statistic (which is a measure of the diversity of the annotations associated with each gene product) since all these singletons have a coherence value equal to one. This situation is clearly visible in Figure 3 where the distributions of the coherence values for "Molecular Function" annotation sets are plotted. The three distributions for AFFY, B2G and AID are bimodal with a first peak between 0.3 and 0.5 and a second one around 1.0 determined mostly by the number of singleton sets. These distributions can be compared to the distribution obtained from a randomly sampled functional annotation where GO terms in the original AFFY annotation sets are replaced by random terms, uniformly drawn from GO. As expected, we see that the original annotation sets from AFFY are much more coherent than the random ones, which confirms that GO terms in AFFY annotation sets tend to be more functionally related than they would otherwise be if drawn at random.

Being able to easily identify and display the annotation sets, where the sizes and/or coherence statistics highlight the presence of extreme values, is a useful feature of AIGO that should help developers of functional annotation pipelines and/or end-users of FAs to detect the presence of abnormalities.

The last three metrics reported in Table 2 are related to the informativeness of the annotations. Both the *specificity *and the *information content *reveal that B2G annotations sets are more informative on average than the two other sets of FAs. We also note the large quantity of redundant annotations present in AFFY and AID annotations sets. For these two FAs, almost 30% of the annotations could potentially be removed from the annotation sets, since they correspond to GO terms which are more generic than the existing terms already present in the same sets. It is however important to note that the GO terms contained in these annotation sets might have been assigned using different techniques with differing levels of confidence. Therefore, when an annotation to a GO term has a stronger confidence than another annotation to a more specific term, it would still make sense to keep both of them together in an annotation set. This may explain the high level of *redundancy *observed in AFFY and AID.

### Inter-comparison of Functional Annotations

**Figure 4:**
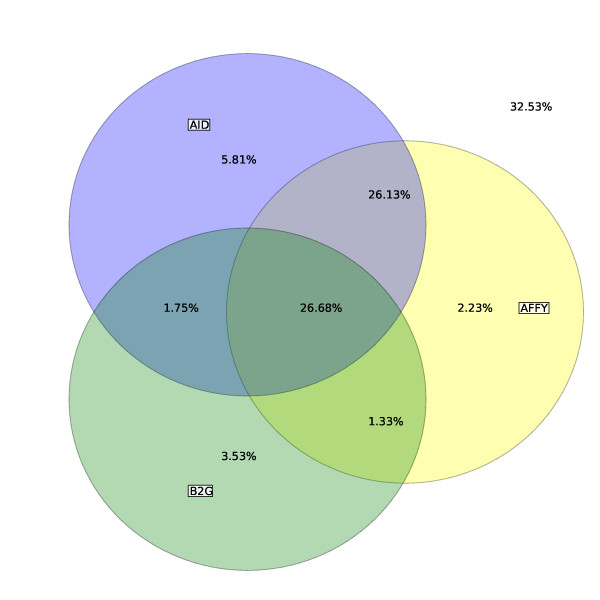
**Coverage of gene products (all aspects of GO) in *Bos taurus***. A Venn diagram comparing the breadth of coverage (in %) of three functional annotations provided by Affymetrix, Blast2GO and ArrayIDer for the Affymetrix GeneChip Bovine Genome Array.

A Venn diagram comparing the breadth of coverage of the three FAs for all three aspects of GO is shown in Figure 4. Altogether AFFY, B2G and AID provide annotations for 67.46% of the set of gene products whilst a subset of only 26.68% is annotated by all the three FAs. Interestingly, we note that a subset of 26.13% of the gene products is annotated by both AFFY and AID but not by B2G. We observe as well that the total difference in coverage between AFFY and B2G or between AID and B2G can be almost entirely explained by this subset. It is beyond the scope of this paper to investigate why these gene products are not annotated by B2G but a similar situation could be reported if, for example, Affymetrix and ArrayIDer pipelines were set up with a less stringent BLAST e-value than B2G or if the Affymetrix and ArrayIDer pipelines were querying a more up-to-date database than B2G, therefore containing more annotated gene products.

**Figure 5:**
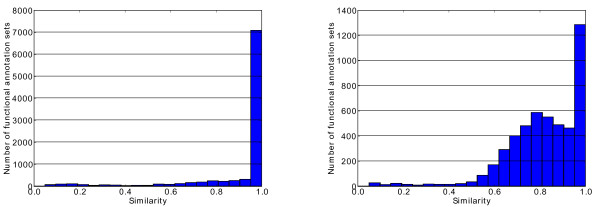
**Semantic similarity scores (all aspects of GO) in *Bos taurus***. Distributions of semantic similarity scores for the Affymetrix GeneChip Bovine Genome Array computed for each gene product commonly annotated by a) Affymetrix and ArrayIDer and b) Affymetrix and Blast2GO.

For the subset that is commonly annotated by the three FAs, it is possible to perform some direct inter-comparisons. For example, Figure 5a) and 5b) display the distributions of the semantic similarity between the annotation sets of the gene products jointly annotated by a) AFFY and AID and b) AFFY and B2G. In this example, the GS2 similarity measure [18] has been used. The annotation sets from AFFY and AID appear to be almost identical for the vast majority of the gene products annotated by both methods, with semantic similarity equals to one. Conversely when comparing AFFY and B2G, we see many more discrepancies between the annotation sets provided by both methods. Several reasons can be suggested to explain the differences between these annotation sets. First of all, they might correspond to situations where gene products perform multiple functions, or are involved in several biological processes or are present in different parts of the cell. Different annotation strategies may lead to different but compatible annotations for a given gene. For example, for the gene MAP2K3 in *Bos taurus*, a sequence-based annotation strategy may recognize the MAPKK3 kinase function and assign the appropriate MAPKKK cascade process (GO:0000165), whereas an approach that uses gene regulation based on microarray expression data might annotate the gene as responsive to stimulus (GO:0050896). However, very different annotation sets will often reveal annotation errors. The AIGO library was used to detect illustrative cases of suspiciously conflicting annotations in the CC aspect of GO. As an example, the probe-set Bt.22320.2.S1_at is annotated to two very different CC GO terms: to "GO:0005634 -nucleus" by AFFY and to "GO:0005576 - extracellular region" by B2G. After verification (see Additional file 3 - Annotations of Bt.22320.2.S1_at), the correct GO term is undoubtedly given by B2G, and the annotation provided by AFFY is clearly an error.

### Example 2: Comparison of evidence codes

The gene association files, which are made available for multiple species by the Gene Ontology project [14], provide mappings of genes to GO terms. For each of these mappings, an evidence code indicates the source from which the association of a given gene with a particular GO term was derived [23]. Only one of these evidence codes, namely "Inferred from Electronic Annotation", identifies annotations that have been made with no manual intervention from a human curator. In this section we use AIGO to compare these electronic annotations to annotations assigned by a human curator, corresponding to the following evidence codes: "Traceable Author Statement" (TAS), "Non-traceable Author Statement" (NAS), "Inferred from sequence or structural similarity" (ISS) and "Inferred by Curator" (IC).

As previously shown [24,25], measuring the accuracy of FAs requires the definition of a reference FA, *i.e. *a gold-standard. Here, we have approximated a gold-standard by identifying all the associations in all the species of the Gene Ontology project that are supported by at least two different experimental-evidence sources based on wet lab experiments. These are represented in GO using the evidence codes "Inferred from Mutant Phenotype", "Inferred from Genetic Interaction", Inferred from Physical Interaction", "Inferred from Direct Assay" and "Inferred from Expression Pattern". All together these associations form a reference FA, which we call EXP2, containing 2,668 experimental annotations related to 1,951 gene products from 20 different species. In a similar way, we have filtered all the associations in all the species to create two other functional annotations: the first one, called IEA, containing only associations corresponding to "Inferred from Electronic Annotation" and the second one, called AHC, containing only associations Assigned by a Human Curator (namely those with evidence codes TAS, NAS, ISS, IC). Approximately 40% of the genes products present in EXP2 are also found in AHC while 89% are also in IEA. For these two subsets of EXP2 gene products, it is therefore possible to compare the annotations in AHC and IEA to the annotations in EXP2 using the *hierarchical precision *(*hPrec*) and the *hierarchical recall *(*hRec*) measures implemented in AIGO. For the three aspects of GO, the average *hPrec *and *hRec *together with the corresponding standard error of the mean are shown in Table 3 and Table 4 respectively.

**Table 3:** Hierarchical Precision of Evidence Codes

Evidence Code	BP	MF	CC
AHC	0.38(0.01)	0.54(0.02)	0.69(0.04)
IEA	0.45(0.01)	0.58(0.02)	0.74(0.02)

Average hierarchical precision (together with the standard error of the mean) of AHC and IEA and EXP2 for the three aspects of GO

**Table 4:** Hierarchical Recall of Evidence Codes

Evidence Code	BP	MF	CC
AHC	0.56(0.02)	0.62(0.02)	0.81(0.04)
IEA	0.53(0.01)	0.82(0.01)	0.81(0.02)

Average hierarchical recall (together with the standard error of the mean) of AHC and IEA and EXP2 for the three aspects of GO

Two important factors influencing these statistics are: for *hRec*, the number of EXP2 annotations to be retrieved and, for *hPrec*, the number of annotations assigned by AHC and IEA. AIGO reveals that the average number of annotations per gene product varies with the GO aspect for the three FAs with: 1.1, 1.15 and 1.2 annotations on average for EXP2 (for respectively CC, MF and BP), 1.67, 1.84 and 4.99 annotations for AHC and 3.56, 4.88 and 6.05 annotations for IEA. The variation reported in the number of annotations for EXP2 explains the trend observed in *hRec *which tends to be smaller for BP than MF and smaller for MF than CC, whereas the variations in AHC and IEA explain the trend for *hPrec *which tends to be smaller for BP than MF and smaller for MF than CC. The relatively low *hPrec *value reported for BP by AHC in Table 3 corresponds to an outstandingly high number of annotations predicted for this category: nearly five annotations per gene product on average, which is more than twice the numbers reported for MF and CC. We note that even though we are confident that EXP2 contains only correct annotations, it is improbable that all the annotations that could be assigned to the gene products are present in EXP2. Hence, the results reported for the *hPrec *metric should be interpreted with care, as incompleteness in the gold-standard may introduce bias into the comparison between FAs.

As for the *hRec *metric, the overall results for AHC and IEA are almost identical, except for MF where IEA performs better. To explore this important difference between IEA and AHC, we looked at the various statistics produced by AIGO for MF. While the *specificity *(mean number of ancestors over all annotated terms) is comparable for AHC and IEA (6.89 and 6.65 respectively), it is apparent that fewer GO terms are used in AHC, compared to IEA and EXP2 (*richness *is 2.82%, 5.26% and 7.97% respectively). The distribution of *hRec *values for AHC displays a peak at 0.2, which is not present for IEA (data not shown). We have manually inspected these cases and found that generally the annotations supported by AHC are also supported by IEA, and are therefore potentially correct. They also tend to correspond to terms from different parts of GO than EXP2 as a whole. We do not further explore this observation in this work but these results reflect the fact that certain parts of the ontology are favoured during the curation process. Indeed, manual associations between GO terms and gene products are often produced as part of manual annotation projects. These curation initiatives tend to have very specific targets such as the annotation of genes related to precise biological functions [26] or to particular diseases [27].

### Example 3: Monitoring the evolution of functional annotations

The AIGO library can also be used to monitor the evolution of FAs across time. As an illustration, AIGO was used to study 11 FAs for a rice genome microarray (see Additional file 1 - Reference of Affymetrix GeneChip genome arrays) released by Affymetrix [6] between 31 May 2007 and 8 November 2010, and numbered from 20 to 31. The set of reference gene products correspond to the 57,381 target sequences of the rice array.

**Figure 6:**
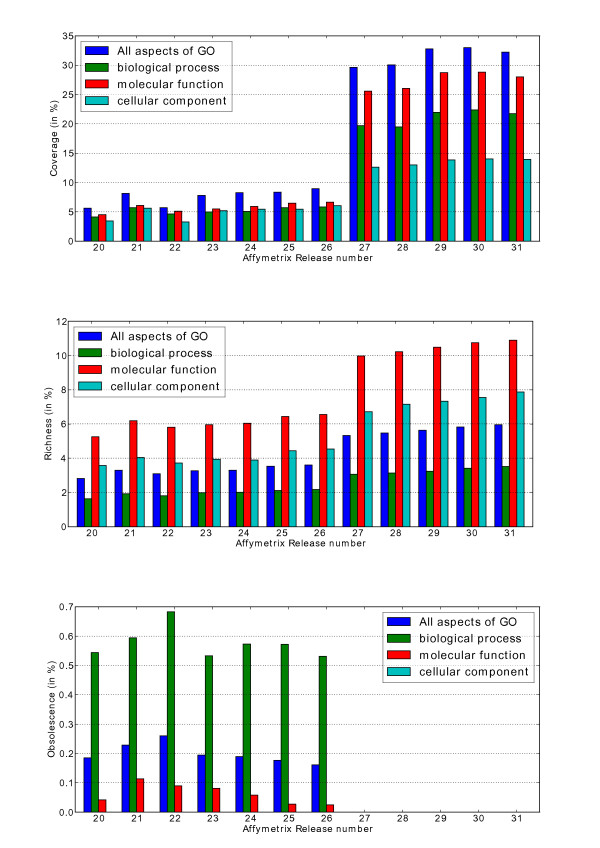
**Evolution of the coverage, obsolescence, and richness of GO annotations (all aspects) in *Oryza sativa***. Evolution of a) the coverage, b) the richness and c) the obsolescence of GO functional annotations for the rice Affymetrix GeneChip genome array. The functional annotations are provided by Affymetrix. They are numbered from 20 to 31 and correspond to 11 releases issued between the 31^st ^May 2007 to the 8 November 2010.

It is clear in Figure 6, that on average, both the number of annotated gene products (*coverage*) and the number of assigned GO terms (*richness*) increase steadily during the studied period of time. However, it is also evident that the coverage can decrease between two releases as was the case between release 21 and 22 for the CC aspect of GO. We note that a major change, both in terms of *coverage *and *richness*, occurred between releases 26 and 27. Affymetrix does not provide any information that would explain this phenomenon, and it could therefore be due to an important step in the evolution in Affymetrix annotation pipeline and/or to a major change in the data sources used to infer the annotations. Interestingly, from release number 27 and onwards, the obsolete annotations have been systematically removed from the FAs, which would suggest that a filtering step has been added to the original Affymetrix annotation pipeline.

**Figure 7:**
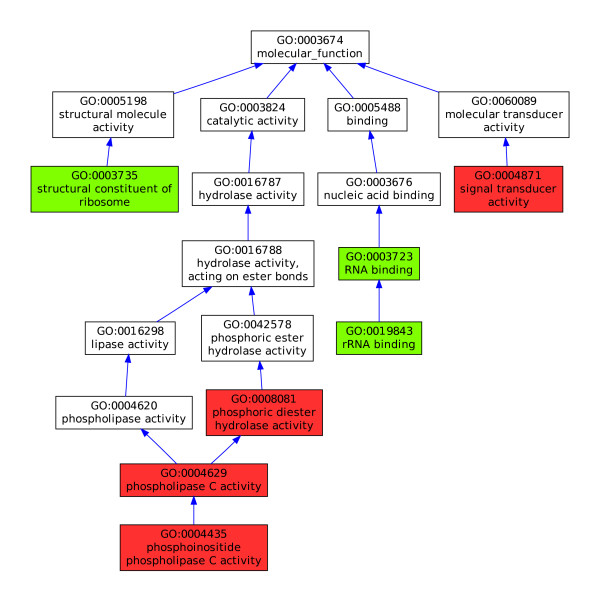
**Two very different sets of annotations provided by Affymetrix between for probe-set Os.2243.1.S1_a_at**. AIGO detected that, in release 20 (green) and in release 31(red), Affymetrix has provided two very different sets of annotation for the probe-set Os.2243.1.S1_a_at. Manual inspection has revealed that the annotations from release 31 are the correct ones.

Affymetrix has issued eleven releases of the rice array annotations since 31 May 2007. During this time, 5% of the probe-sets have been annotated in every release and it is therefore possible to study the evolution of the annotation sets for the corresponding probe-sets. In Figure 7, AIGO was used to identify and plot two very dissimilar annotation sets found in release 20 (green) and release 31(red) for the probe-set Os.2243.1.S1_a_at. In this example, the sets have no annotation in common and when considering the structure of the ontology, they appear to be far apart in the GO hierarchy, corresponding to a very low GS2 similarity (≈0.2). If we manually examine the two annotation sets from a biological perspective, they also appear to be functionally incompatible. In fact, there is only one unique example (See Additional file 4 - Rice protein with suspicious annotations) in the entire Gene Ontology Annotation Database where the two GO terms "structural constituent of ribosome" and "phospholipase C activity" have been used together to describe a single protein. Therefore, we can assume that the difference between the two annotation sets does not correspond to a natural evolution of the functional annotation over time, for example, due to an improvement in the description of the function of the protein associated with Os.2243.1.S1_a_at, but rather to an important correction of an original error made in the annotation process. To gain further insight into the annotation of Os.2243.1.S1_a_at, the complementary information provided by Affymetrix was analysed in more detail. It appears that two different gene loci in *Oryza sativa *(both on chromosome 5, but at different locations) were used to infer this annotation. After comparing the target sequence Os.2243.1.S1_a_at, with the sequences of the proteins encoded by these two different genes, we have been able to confirm that the annotations from release 31 are correct (see Additional file 5 - Annotations of Os.2243.1.S1_a_at).

## Discussion

Biologists and bioinformaticians, as end-users of functional annotations (FAs), are generally confronted with a choice between several available FAs for the organisms they are studying. Often the most important thing to consider when deciding between alternative FAs is to look at the accuracy of the annotations: *i.e. *to what extent do the annotations of some proteins really correspond to what has been experimentally established about the function of these proteins? In practice this evaluation is very challenging, given that for the vast majority of the organisms there is no existing gold-standard to evaluate precision and recall against. Furthermore, even in the rare cases where an organism has been extensively studied and the functions of most of its proteins experimentally assessed and annotated in public databases, there is no guarantee that all the biological roles and functions of these proteins are known and translated into functional annotations. This situation is problematic when evaluating the *precision *of FAs, but is not unique to the study of protein function since the lack of negative gold-standards and its impact on the evaluation of automatic inference methods has been reported in other domains of bioinformatics [28].

An alternative approach to decide between FAs would be to directly compare the functional annotation pipelines that produced these FAs. However, these pipelines differ widely in their inference algorithms, confidence thresholds, and data sources for reasoning and this makes the comparison of the relative merits of each approach extremely complex.

The more pragmatic approach developed in this paper is to analyse and inter-compare the products of these functional annotation pipelines, that is the FAs themselves. FAs are complex multi-dimensional objects that cannot be summarized in a straightforward way. Hence, we defined the AIGO framework to describe various features of FAs that we consider being important for FAs end-users and more generally for the community of biologists and bioinformaticians interested in gene or protein functions. So far a set of twelve metrics, nine for the analysis and three for the inter-comparison of FAs, has been defined and implemented in AIGO. Each metric covers only one particular aspect of the FAs but, as demonstrated in this paper, when computed all together and interpreted in the AIGO framework, these metrics provide a global view of the FAs and help to make informed decisions concerning them. Alternatively, after having inspected a set of FAs in AIGO, one might want to combine them instead of retaining only one. Therefore, the library also provides functionalities to compute the union or the intersection of FAs. The union of FAs is, for example, particularly meaningful in the case where each FA covers a different aspect of GO, *e.g. *to combine a FA containing only GO Molecular Function terms converted from Enzyme Commission numbers with a FA containing only GO Cellular Component terms converted from subcellular location predictions. Conversely, the intersection of FAs is more appropriate to increase the reliability of functional annotations. For example, when an ensemble of FAs provides annotations about the same organism but using different types of evidence (*e.g.*, sequence or structural comparisons, analysis of gene coexpression patterns, etc.) selecting the associations between gene products and GO terms that are present in all these FAs is a straightforward way to gain confidence in the annotations.

As well as FA end-users, we believe that AIGO can be beneficial to other categories of members of the scientific community. An important category of members corresponds to FA producers, *i.e. *people developing new functional annotation pipelines, who might want to compare their pipeline outputs to existing FAs, or measure the influence of a given pipeline parameter on their results. Another category is the providers of FAs who are responsible for releasing FAs to the community, and who need periodically to monitor the content of automatically produced FAs, for example, to detect spurious annotations or abnormally large annotation sets. A further category corresponds to researchers working on new metrics to describe FAs. The AIGO framework has been designed to be flexible so that new metrics on GO functional annotations can be easily added. By making this project open source and collaborative, we would like to encourage researchers to implement new metrics in AIGO and contribute to its development.

## Conclusions

The work presented in this paper is a first step towards the development of a unified framework for the analysis and the inter-comparison of GO functional annotations.

The utility of the framework is demonstrated on three case studies. In the first, publicly available functional annotations are compared, and their differences highlighted, for example, in terms of the number of gene products annotated, or the number and specificity of the GO terms employed. In the second case study, the quality of two functional annotations, one obtained by computational methods and one corresponding to a manual curation process, is assessed using an approximated gold-standard. In the last example, we show how AIGO framework can be used to monitor the evolution of functional annotations by comparing different releases over time, in order to detect major variations, or to identify potentially incorrect annotations.

## Authors' contributions

MDP initiated the work, developed the AIGO library, designed the tests, carried out all the functional annotation studies and drafted the manuscript. MH was involved in the design of the experiments, contributed to the development of the library and participated in redaction of the manuscript. AL created the gold-standard dataset for Example 2. DZH, CJR and MS contributed to the coordination of the studies and helped to draft the manuscript. All authors read and approved the final manuscript.

## Supplementary Material

Additional file 1 **Reference of Affymetrix GeneChip genome arrays**. Click here for file

Additional file 2 **Annotations of Bt.13141.1.S1_at**. Click here for file

Additional file 3 **Annotations of Bt.22320.2.S1_at**. Click here for file

Additional file 4 **Rice protein with suspicious annotations**. Click here for file

Additional file 5 **Annotations of Os.2243.1.S1_a_at**. Click here for file

## References

[B1] Schmid R Blaxter ML annot8r: GO, EC and KEGG annotation of EST datasets BMC Bioinformatics 2008 9 180 10.1186/1471-2105-9-180 18400082 PMC2324097

[B2] Huang DW Sherman BT Tan Q Collins JR Alvord WG Roayaei J Stephens R Baseler MW Lane HC Lempicki RA The DAVID Gene Functional Classification Tool: a novel biological module-centric algorithm to functionally analyze large gene lists Genome Biology 2007 8 9 R183 10.1186/gb-2007-8-9-r183 17784955 PMC2375021

[B3] Koski LB Gray MW Lang BF Burger G AutoFACT: An (Auto)matic (F)unctional (A)nnotation and (C)lassification (T)ool BMC Bioinformatics 2005 6 151 10.1186/1471-2105-6-151 15960857 PMC1182349

[B4] Conesa A Götz S Garcia-Gomez JM Terol J Talon M Robles M Blast2GO: a universal tool for annotation, visualization and analysis in functional genomics research Bioinformatics 2005 21 18 3674 3676 10.1093/bioinformatics/bti610 16081474

[B5] Martin DMA Berriman M Barton GJ GOtcha: a new method for prediction of protein function assessed by the annotation of seven genomes BMC Bioinformatics 2004 5 178 10.1186/1471-2105-5-178 15550167 PMC535938

[B6] Liu GY Loraine AE Shigeta R Cline M Cheng J Valmeekam V Sun S Kulp D Siani-Rose MA NetAffx: Affymetrix probesets and annotations Nucleic Acids Research 2003 31 1 82 86 10.1093/nar/gkg121 12519953 PMC165568

[B7] The Gene Ontology Consortium Creating the gene ontology resource: design and implementation Genome Research 2001 11 8 1425 1433 10.1101/gr.180801 11483584 PMC311077

[B8] Conesa A Götz S Blast2GO: A Comprehensive Suite for Functional Analysis in Plant Genomics International journal of plant genomics 2008 2008 619832 619832 18483572 10.1155/2008/619832 PMC2375974

[B9] Chagoyen M Carazo JM Pascual-Montano A Assessment of protein set coherence using functional annotations BMC Bioinformatics 2008 9 444 10.1186/1471-2105-9-444 18937846 PMC2588600

[B10] Buza TJ McCarthy FM Wang N Bridges SM Burgess SC Gene Ontology annotation quality analysis in model eukaryotes Nucleic Acids Research 2008 36 2 e12 18187504 10.1093/nar/gkm1167 PMC2241866

[B11] Wheeler DL Church DM Federhen S Lash AE Madden TL Pontius JU Schuler GD Schriml LM Sequeira E Tatusova TA Wagner L Database resources of the National Center for Biotechnology Nucleic Acids Research 2003 31 1 28 33 10.1093/nar/gkg033 12519941 PMC165480

[B12] Guo X Liu RX Shriver CD Hu H Liebman MN Assessing semantic similarity measures for the characterization of human regulatory pathways Bioinformatics 2006 22 8 967 973 10.1093/bioinformatics/btl042 16492685

[B13] Pesquita C Faria D Bastos H Ferreira AE Falcao AO Couto FM Metrics for GO based protein semantic similarity: a systematic evaluation BMC Bioinformatics 2008 9 Suppl 5 S4 10.1186/1471-2105-9-S5-S4 18460186 PMC2367622

[B14] Gene Ontology Consortium The Gene Ontology's Reference Genome Project: a unified framework for functional annotation across species PLoS Computational Biology 2009 5 7 e1000431 10.1371/journal.pcbi.1000431 19578431 PMC2699109

[B15] Shannon CE A Mathematical Theory of Communication Bell System Technical Journal 1948 27 3 379 423

[B16] Resnik P Using information content to evaluate semantic similarity in a taxonomy Proceedings of the 14th International Joint Conference on Artificial Intelligence 1995 1 448 453

[B17] Brun C Chevenet F Martin D Wojcik J Guenoche A Jacq B Functional classification of proteins for the prediction of cellular function from a protein-protein interaction network Genome Biology 2003 5 1 R6 10.1186/gb-2003-5-1-r6 14709178 PMC395738

[B18] Ruths T Ruths D Nakhleh L GS2: an efficiently computable measure of GO-based similarity of gene sets Bioinformatics (Oxford, England) 2009 25 9 1178 1184 10.1093/bioinformatics/btp128 PMC2672633 19289444

[B19] Wang JZ Du Z Payattakool R Yu PS Chen CF A new method to measure the semantic similarity of GO terms Bioinformatics (Oxford, England) 2007 23 10 1274 1281 10.1093/bioinformatics/btm087 17344234

[B20] Verspoor K Cohn J Mniszewski S Joslyn C A categorization approach to automated ontological function annotation Protein Science 2006 15 6 1544 1549 10.1110/ps.062184006 16672243 PMC2242540

[B21] Götz S Arnold R Sebastián-León P Martín-Rodríguez S Tischler P Jehl MA Dopazo J Rattei T Conesa A B2G-FAR, a species centered GO annotation repository Bioinformatics 2011 27 7 919 924 10.1093/bioinformatics/btr059 21335611 PMC3065692

[B22] van den Berg BH Konieczka JH McCarthy FM Burgess SC ArrayIDer: automated structural re-annotation pipeline for DNA microarrays BMC Bioinformatics 2009 10 30 10.1186/1471-2105-10-30 19166590 PMC2636773

[B23] Guide to GO Evidence Codes http://www.geneontology.org/GO.evidence.shtml

[B24] Pal D Eisenberg D Inference of Protein Function from Protein Structure Structure 2005 13 1 121 130 10.1016/j.str.2004.10.015 15642267

[B25] Verspoor K Cohn J Mniszewski S Joslyn C A categorization approach to automated ontological function annotation Protein Sci 2006 15 6 1544 1549 10.1110/ps.062184006 16672243 PMC2242540

[B26] Alam-Faruque Y Dimmer EC Huntley RP O'Donovan C Scambler P Apweiler R The Renal Gene Ontology Annotation Initiative Organogenesis 2010 6 2 71 75 10.4161/org.6.2.11294 20885853 PMC2901810

[B27] Khodiyar VK Hill DP Howe D Berardini TZ Tweedie S Talmud PJ Breckenridge R Bhattarcharya S Riley P Scambler P Lovering RC The representation of heart development in the gene ontology Developmental Biology 2011 354 1 9 17 10.1016/j.ydbio.2011.03.011 21419760 PMC3302178

[B28] Jansen R Gerstein M Analyzing protein function on a genomic scale: the importance of gold-standard positives and negatives for network prediction Current opinion in microbiology 2004 7 5 535 545 10.1016/j.mib.2004.08.012 15451510

