# Supplementary material for: AIGO: Towards a unified framework for the Analysis and the Inter-comparison of GO functional annotations
Source: BMC Bioinformatics. 2011 Nov 3;12:431. doi: 10.1186/1471-2105-12-431 (PMC3237112; doi:10.1186/1471-2105-12-431)
Supplement: Additional file 1 — Reference of Affymetrix GeneChip genome arrays. [file 1471-2105-12-431-S1.DOC]

### Reference of Affymetrix GeneChip genome arrays

Reference of the Bovine array:

[http://www.affymetrix.com/browse/products.jsp?productId=131421&navMode=34000&navAction=jump&aId=productsNav#1_3](http://www.affymetrix.com/browse/products.jsp?productId=131421&navMode=34000&navAction=jump&aId=productsNav#1_3)

Reference of the Rice array:

[http://www.affymetrix.com/browse/products.jsp?productId=131497&navMode=34000&navAction=jump&aId=productsNav#1_3](http://www.affymetrix.com/browse/products.jsp?productId=131497&navMode=34000&navAction=jump&aId=productsNav#1_3)
