# Supplementary material for: AIGO: Towards a unified framework for the Analysis and the Inter-comparison of GO functional annotations
Source: BMC Bioinformatics. 2011 Nov 3;12:431. doi: 10.1186/1471-2105-12-431 (PMC3237112; doi:10.1186/1471-2105-12-431)
Supplement: Additional file 2 — Annotations of Bt.13141.1.S1_at. [file 1471-2105-12-431-S2.DOC]

### Annotations of Bt.13141.1.S1_at

!Annotation statistics

!Downloaded from QuickGO at 2011-04-01 12:08

!URL: http://www.ebi.ac.uk/QuickGO/GAnnotation?count=1000&protein=Q462R3&select=normal&advanced=&termUse=ancestor&slimTypes=IP%3D

!Contact email: goa@ebi.ac.uk

!

[Summary]

Number of annotations Number of distinct proteins

164 1

[GO IDs (by annotation)]

Code Name Percentage Count

GO:0042981 regulation of apoptosis 3.05 5

GO:0046982 protein heterodimerization activity 1.22 2

GO:0043524 negative regulation of neuron apoptosis 1.22 2

GO:0043066 negative regulation of apoptosis 1.22 2

GO:0042493 response to drug 1.22 2

GO:0034097 response to cytokine stimulus 1.22 2

GO:0031965 nuclear membrane 1.22 2

GO:0016563 transcription activator activity 1.22 2

GO:0009636 response to toxin 1.22 2

GO:0008219 cell death 1.22 2

GO:0006916 anti-apoptosis 1.22 2

GO:0005739 mitochondrion 1.22 2

GO:0005737 cytoplasm 1.22 2

GO:0005634 nucleus 1.22 2

GO:0070059 apoptosis in response to endoplasmic reticulum stress 0.61 1

GO:0051924 regulation of calcium ion transport 0.61 1

GO:0051881 regulation of mitochondrial membrane potential 0.61 1

GO:0051789 response to protein stimulus 0.61 1

GO:0051726 regulation of cell cycle 0.61 1

GO:0051721 protein phosphatase 2A binding 0.61 1

GO:0051607 defense response to virus 0.61 1

GO:0051434 BH3 domain binding 0.61 1

GO:0051402 neuron apoptosis 0.61 1

GO:0051384 response to glucocorticoid stimulus 0.61 1

GO:0050853 B cell receptor signaling pathway 0.61 1

GO:0050790 regulation of catalytic activity 0.61 1

GO:0048873 homeostasis of number of cells within a tissue 0.61 1

GO:0048753 pigment granule organization 0.61 1

GO:0048743 positive regulation of skeletal muscle fiber development 0.61 1

GO:0048599 oocyte development 0.61 1

GO:0048589 developmental growth 0.61 1

GO:0048546 digestive tract morphogenesis 0.61 1

GO:0048545 response to steroid hormone stimulus 0.61 1

GO:0048538 thymus development 0.61 1

GO:0048536 spleen development 0.61 1

GO:0048087 positive regulation of developmental pigmentation 0.61 1

GO:0048070 regulation of developmental pigmentation 0.61 1

GO:0048066 developmental pigmentation 0.61 1

GO:0048041 focal adhesion assembly 0.61 1

GO:0046902 regulation of mitochondrial membrane permeability 0.61 1

GO:0046671 negative regulation of retinal cell programmed cell death 0.61 1

GO:0045930 negative regulation of mitotic cell cycle 0.61 1

GO:0045636 positive regulation of melanocyte differentiation 0.61 1

GO:0045069 regulation of viral genome replication 0.61 1

GO:0043583 ear development 0.61 1

GO:0043565 sequence-specific DNA binding 0.61 1

GO:0043497 regulation of protein heterodimerization activity 0.61 1

GO:0043496 regulation of protein homodimerization activity 0.61 1

GO:0043473 pigmentation 0.61 1

GO:0043375 CD8-positive, alpha-beta T cell lineage commitment 0.61 1

GO:0043209 myelin sheath 0.61 1

GO:0043085 positive regulation of catalytic activity 0.61 1

GO:0043067 regulation of programmed cell death 0.61 1

GO:0043029 T cell homeostasis 0.61 1

GO:0042803 protein homodimerization activity 0.61 1

GO:0042802 identical protein binding 0.61 1

GO:0042542 response to hydrogen peroxide 0.61 1

GO:0042221 response to chemical stimulus 0.61 1

GO:0042100 B cell proliferation 0.61 1

GO:0040018 positive regulation of multicellular organism growth 0.61 1

GO:0040007 growth 0.61 1

GO:0035265 organ growth 0.61 1

GO:0035094 response to nicotine 0.61 1

GO:0033689 negative regulation of osteoblast proliferation 0.61 1

GO:0033138 positive regulation of peptidyl-serine phosphorylation 0.61 1

GO:0033077 T cell differentiation in thymus 0.61 1

GO:0033033 negative regulation of myeloid cell apoptosis 0.61 1

GO:0032880 regulation of protein localization 0.61 1

GO:0032848 negative regulation of cellular pH reduction 0.61 1

GO:0032835 glomerulus development 0.61 1

GO:0032469 endoplasmic reticulum calcium ion homeostasis 0.61 1

GO:0031966 mitochondrial membrane 0.61 1

GO:0031647 regulation of protein stability 0.61 1

GO:0031103 axon regeneration 0.61 1

GO:0031069 hair follicle morphogenesis 0.61 1

GO:0030890 positive regulation of B cell proliferation 0.61 1

GO:0030336 negative regulation of cell migration 0.61 1

GO:0030318 melanocyte differentiation 0.61 1

GO:0030308 negative regulation of cell growth 0.61 1

GO:0030307 positive regulation of cell growth 0.61 1

GO:0030279 negative regulation of ossification 0.61 1

GO:0030217 T cell differentiation 0.61 1

GO:0030183 B cell differentiation 0.61 1

GO:0030097 hemopoiesis 0.61 1

GO:0022612 gland morphogenesis 0.61 1

GO:0021747 cochlear nucleus development 0.61 1

GO:0019903 protein phosphatase binding 0.61 1

GO:0018107 peptidyl-threonine phosphorylation 0.61 1

GO:0018105 peptidyl-serine phosphorylation 0.61 1

GO:0016337 cell-cell adhesion 0.61 1

GO:0016049 cell growth 0.61 1

GO:0016020 membrane 0.61 1

GO:0014911 positive regulation of smooth muscle cell migration 0.61 1

GO:0014042 positive regulation of neuron maturation 0.61 1

GO:0014031 mesenchymal cell development 0.61 1

GO:0010559 regulation of glycoprotein biosynthetic process 0.61 1

GO:0010523 negative regulation of calcium ion transport into cytosol 0.61 1

GO:0010468 regulation of gene expression 0.61 1

GO:0010332 response to gamma radiation 0.61 1

GO:0010224 response to UV-B 0.61 1

GO:0010039 response to iron ion 0.61 1

GO:0009887 organ morphogenesis 0.61 1

GO:0009791 post-embryonic development 0.61 1

GO:0009605 response to external stimulus 0.61 1

GO:0008584 male gonad development 0.61 1

GO:0008285 negative regulation of cell proliferation 0.61 1

GO:0008284 positive regulation of cell proliferation 0.61 1

GO:0008283 cell proliferation 0.61 1

GO:0008134 transcription factor binding 0.61 1

GO:0007569 cell aging 0.61 1

GO:0007409 axonogenesis 0.61 1

GO:0007015 actin filament organization 0.61 1

GO:0006979 response to oxidative stress 0.61 1

GO:0006974 response to DNA damage stimulus 0.61 1

GO:0006915 apoptosis 0.61 1

GO:0006874 cellular calcium ion homeostasis 0.61 1

GO:0006808 regulation of nitrogen utilization 0.61 1

GO:0006582 melanin metabolic process 0.61 1

GO:0006470 protein dephosphorylation 0.61 1

GO:0005829 cytosol 0.61 1

GO:0005792 microsome 0.61 1

GO:0005789 endoplasmic reticulum membrane 0.61 1

GO:0005783 endoplasmic reticulum 0.61 1

GO:0005624 membrane fraction 0.61 1

GO:0005622 intracellular 0.61 1

GO:0003014 renal system process 0.61 1

GO:0002520 immune system development 0.61 1

GO:0002360 T cell lineage commitment 0.61 1

GO:0002326 B cell lineage commitment 0.61 1

GO:0002320 lymphoid progenitor cell differentiation 0.61 1

GO:0002260 lymphocyte homeostasis 0.61 1

GO:0002020 protease binding 0.61 1

GO:0001952 regulation of cell-matrix adhesion 0.61 1

GO:0001836 release of cytochrome c from mitochondria 0.61 1

GO:0001822 kidney development 0.61 1

GO:0001782 B cell homeostasis 0.61 1

GO:0001776 leukocyte homeostasis 0.61 1

GO:0001662 behavioral fear response 0.61 1

GO:0001658 branching involved in ureteric bud morphogenesis 0.61 1

GO:0001657 ureteric bud development 0.61 1

GO:0001656 metanephros development 0.61 1

GO:0001541 ovarian follicle development 0.61 1

GO:0001503 ossification 0.61 1

GO:0001101 response to acid 0.61 1

GO:0000902 cell morphogenesis 0.61 1

GO:0000209 protein polyubiquitination 0.61 1

GO:0000082 G1/S transition of mitotic cell cycle 0.61 1

[GO IDs (by protein)]

Code Name Percentage Count

GO:0070059 apoptosis in response to endoplasmic reticulum stress 100.00 1

GO:0051924 regulation of calcium ion transport 100.00 1

GO:0051881 regulation of mitochondrial membrane potential 100.00 1

GO:0051789 response to protein stimulus 100.00 1

GO:0051726 regulation of cell cycle 100.00 1

GO:0051721 protein phosphatase 2A binding 100.00 1

GO:0051607 defense response to virus 100.00 1

GO:0051434 BH3 domain binding 100.00 1

GO:0051402 neuron apoptosis 100.00 1

GO:0051384 response to glucocorticoid stimulus 100.00 1

GO:0050853 B cell receptor signaling pathway 100.00 1

GO:0050790 regulation of catalytic activity 100.00 1

GO:0048873 homeostasis of number of cells within a tissue 100.00 1

GO:0048753 pigment granule organization 100.00 1

GO:0048743 positive regulation of skeletal muscle fiber development 100.00 1

GO:0048599 oocyte development 100.00 1

GO:0048589 developmental growth 100.00 1

GO:0048546 digestive tract morphogenesis 100.00 1

GO:0048545 response to steroid hormone stimulus 100.00 1

GO:0048538 thymus development 100.00 1

GO:0048536 spleen development 100.00 1

GO:0048087 positive regulation of developmental pigmentation 100.00 1

GO:0048070 regulation of developmental pigmentation 100.00 1

GO:0048066 developmental pigmentation 100.00 1

GO:0048041 focal adhesion assembly 100.00 1

GO:0046982 protein heterodimerization activity 100.00 1

GO:0046902 regulation of mitochondrial membrane permeability 100.00 1

GO:0046671 negative regulation of retinal cell programmed cell death 100.00 1

GO:0045930 negative regulation of mitotic cell cycle 100.00 1

GO:0045636 positive regulation of melanocyte differentiation 100.00 1

GO:0045069 regulation of viral genome replication 100.00 1

GO:0043583 ear development 100.00 1

GO:0043565 sequence-specific DNA binding 100.00 1

GO:0043524 negative regulation of neuron apoptosis 100.00 1

GO:0043497 regulation of protein heterodimerization activity 100.00 1

GO:0043496 regulation of protein homodimerization activity 100.00 1

GO:0043473 pigmentation 100.00 1

GO:0043375 CD8-positive, alpha-beta T cell lineage commitment 100.00 1

GO:0043209 myelin sheath 100.00 1

GO:0043085 positive regulation of catalytic activity 100.00 1

GO:0043067 regulation of programmed cell death 100.00 1

GO:0043066 negative regulation of apoptosis 100.00 1

GO:0043029 T cell homeostasis 100.00 1

GO:0042981 regulation of apoptosis 100.00 1

GO:0042803 protein homodimerization activity 100.00 1

GO:0042802 identical protein binding 100.00 1

GO:0042542 response to hydrogen peroxide 100.00 1

GO:0042493 response to drug 100.00 1

GO:0042221 response to chemical stimulus 100.00 1

GO:0042100 B cell proliferation 100.00 1

GO:0040018 positive regulation of multicellular organism growth 100.00 1

GO:0040007 growth 100.00 1

GO:0035265 organ growth 100.00 1

GO:0035094 response to nicotine 100.00 1

GO:0034097 response to cytokine stimulus 100.00 1

GO:0033689 negative regulation of osteoblast proliferation 100.00 1

GO:0033138 positive regulation of peptidyl-serine phosphorylation 100.00 1

GO:0033077 T cell differentiation in thymus 100.00 1

GO:0033033 negative regulation of myeloid cell apoptosis 100.00 1

GO:0032880 regulation of protein localization 100.00 1

GO:0032848 negative regulation of cellular pH reduction 100.00 1

GO:0032835 glomerulus development 100.00 1

GO:0032469 endoplasmic reticulum calcium ion homeostasis 100.00 1

GO:0031966 mitochondrial membrane 100.00 1

GO:0031965 nuclear membrane 100.00 1

GO:0031647 regulation of protein stability 100.00 1

GO:0031103 axon regeneration 100.00 1

GO:0031069 hair follicle morphogenesis 100.00 1

GO:0030890 positive regulation of B cell proliferation 100.00 1

GO:0030336 negative regulation of cell migration 100.00 1

GO:0030318 melanocyte differentiation 100.00 1

GO:0030308 negative regulation of cell growth 100.00 1

GO:0030307 positive regulation of cell growth 100.00 1

GO:0030279 negative regulation of ossification 100.00 1

GO:0030217 T cell differentiation 100.00 1

GO:0030183 B cell differentiation 100.00 1

GO:0030097 hemopoiesis 100.00 1

GO:0022612 gland morphogenesis 100.00 1

GO:0021747 cochlear nucleus development 100.00 1

GO:0019903 protein phosphatase binding 100.00 1

GO:0018107 peptidyl-threonine phosphorylation 100.00 1

GO:0018105 peptidyl-serine phosphorylation 100.00 1

GO:0016563 transcription activator activity 100.00 1

GO:0016337 cell-cell adhesion 100.00 1

GO:0016049 cell growth 100.00 1

GO:0016020 membrane 100.00 1

GO:0014911 positive regulation of smooth muscle cell migration 100.00 1

GO:0014042 positive regulation of neuron maturation 100.00 1

GO:0014031 mesenchymal cell development 100.00 1

GO:0010559 regulation of glycoprotein biosynthetic process 100.00 1

GO:0010523 negative regulation of calcium ion transport into cytosol 100.00 1

GO:0010468 regulation of gene expression 100.00 1

GO:0010332 response to gamma radiation 100.00 1

GO:0010224 response to UV-B 100.00 1

GO:0010039 response to iron ion 100.00 1

GO:0009887 organ morphogenesis 100.00 1

GO:0009791 post-embryonic development 100.00 1

GO:0009636 response to toxin 100.00 1

GO:0009605 response to external stimulus 100.00 1

GO:0008584 male gonad development 100.00 1

GO:0008285 negative regulation of cell proliferation 100.00 1

GO:0008284 positive regulation of cell proliferation 100.00 1

GO:0008283 cell proliferation 100.00 1

GO:0008219 cell death 100.00 1

GO:0008134 transcription factor binding 100.00 1

GO:0007569 cell aging 100.00 1

GO:0007409 axonogenesis 100.00 1

GO:0007015 actin filament organization 100.00 1

GO:0006979 response to oxidative stress 100.00 1

GO:0006974 response to DNA damage stimulus 100.00 1

GO:0006916 anti-apoptosis 100.00 1

GO:0006915 apoptosis 100.00 1

GO:0006874 cellular calcium ion homeostasis 100.00 1

GO:0006808 regulation of nitrogen utilization 100.00 1

GO:0006582 melanin metabolic process 100.00 1

GO:0006470 protein dephosphorylation 100.00 1

GO:0005829 cytosol 100.00 1

GO:0005792 microsome 100.00 1

GO:0005789 endoplasmic reticulum membrane 100.00 1

GO:0005783 endoplasmic reticulum 100.00 1

GO:0005739 mitochondrion 100.00 1

GO:0005737 cytoplasm 100.00 1

GO:0005634 nucleus 100.00 1

GO:0005624 membrane fraction 100.00 1

GO:0005622 intracellular 100.00 1

GO:0003014 renal system process 100.00 1

GO:0002520 immune system development 100.00 1

GO:0002360 T cell lineage commitment 100.00 1

GO:0002326 B cell lineage commitment 100.00 1

GO:0002320 lymphoid progenitor cell differentiation 100.00 1

GO:0002260 lymphocyte homeostasis 100.00 1

GO:0002020 protease binding 100.00 1

GO:0001952 regulation of cell-matrix adhesion 100.00 1

GO:0001836 release of cytochrome c from mitochondria 100.00 1

GO:0001822 kidney development 100.00 1

GO:0001782 B cell homeostasis 100.00 1

GO:0001776 leukocyte homeostasis 100.00 1

GO:0001662 behavioral fear response 100.00 1

GO:0001658 branching involved in ureteric bud morphogenesis 100.00 1

GO:0001657 ureteric bud development 100.00 1

GO:0001656 metanephros development 100.00 1

GO:0001541 ovarian follicle development 100.00 1

GO:0001503 ossification 100.00 1

GO:0001101 response to acid 100.00 1

GO:0000902 cell morphogenesis 100.00 1

GO:0000209 protein polyubiquitination 100.00 1

GO:0000082 G1/S transition of mitotic cell cycle 100.00 1

[Evidence Codes (by annotation)]

Code Percentage Count

IEA 100.00 164

[Evidence Codes (by protein)]

Code Percentage Count

IEA 100.00 1

[Qualifiers (by annotation)]

Code Percentage Count

(none) 100.00 164

[Qualifiers (by protein)]

Code Percentage Count

(none) 100.00 1

[Sources (by annotation)]

Code Percentage Count

ENSEMBL 97.56 160

InterPro 2.44 4

[Sources (by protein)]

Code Percentage Count

ENSEMBL 100.00 1

InterPro 100.00 1

[Taxon IDs (by annotation)]

Code Name Percentage Count

9913 Bos taurus 100.00 164

[Taxon IDs (by protein)]

Code Name Percentage Count

9913 Bos taurus 100.00 1
