# Supplementary material for: AIGO: Towards a unified framework for the Analysis and the Inter-comparison of GO functional annotations
Source: BMC Bioinformatics. 2011 Nov 3;12:431. doi: 10.1186/1471-2105-12-431 (PMC3237112; doi:10.1186/1471-2105-12-431)
Supplement: Additional file 3 — Annotations of Bt.22320.2.S1_at. [file 1471-2105-12-431-S3.DOC]

### Annotations of Bt.13141.1.S1_at

To better understand the conflicting annotations reported by AFFY and B2G, we used BLASTx to compare the target sequence of Bt.22320.2.S1_at, as provided by Affymetrix, to the NR database. A match (Identities = 86%, Positives = 91%, see below) to a serine peptidase, mannan-binding lectin serine peptidase 2 (MASP2) in *Bos Taurus* (**E1BJ49) is reported**, for which no information about cellular localisation is yet available in Uniprot. However, the MASP2 comprises several members and the corresponding protein in Human (**O00187**) has been experimentally identified as a secreted protein. The evidence code of its CC annotation is consequently “extracellular region” (source Reactome). This result confirms that the annotation produced by B2G is the correct one. After inspecting the Affymetrix annotation file, we found that it referred to a PIR record (A59271) which is the Human MASP2 protein, but that it also referred to a TAR DNA binding protein (Q2KJ45) which has clearly no sequence similarity with Bt.22320.2.S1_at, but was nonetheless used to infer its annotation.

>[ref|XP_002694103.1|](http://www.ncbi.nlm.nih.gov/protein/297484098?report=genbank&log$=protalign&blast_rank=1&RID=M41XX59D014) [
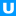
](http://www.ncbi.nlm.nih.gov/entrez/query.fcgi?db=protein&cmd=Display&dopt=protein_unigene&from_uid=297484098&RID=M41XX59D014&log$=unigenealign&blast_rank=1)[
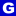
](http://www.ncbi.nlm.nih.gov/entrez/query.fcgi?db=gene&cmd=search&term=297484098[PUID]&RID=M41XX59D014&log$=genealign&blast_rank=1)[
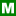
](http://www.ncbi.nlm.nih.gov/mapview/map_search.cgi?direct=on&gbgi=297484098&THE_BLAST_RID=M41XX59D014&log$=mapalign&blast_rank=1) PREDICTED: mannan-binding lectin serine peptidase 2 [Bos taurus]

[gb|DAA21313.1|](http://www.ncbi.nlm.nih.gov/protein/296479198?report=genbank&log$=protalign&blast_rank=1&RID=M41XX59D014) [
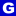
](http://www.ncbi.nlm.nih.gov/entrez/query.fcgi?db=gene&cmd=search&term=296479198[PUID]&RID=M41XX59D014&log$=genealign&blast_rank=1) mannan-binding lectin serine peptidase 2 [Bos taurus]

Length=441

[GENE ID: 505819 MASP2](http://www.ncbi.nlm.nih.gov/sites/entrez?db=gene&cmd=search&term=505819&RID=M41XX59D014&log$=geneexplicitprot&blast_rank=1) | mannan-binding lectin serine peptidase 2 [Bos taurus]

(10 or fewer PubMed links)

Score = 102 bits (254), Expect = 2e-20

Identities = 46/54 (86%), Positives = 49/54 (91%), Gaps = 0/54 (0%)

Frame = -1

Query 487 VVDCGPPDDLPSGQVEYITGPAVTTYRAVVKYRCNEFYTMTTNDGKQTVPSWGW 326

+VDCGPPDDLPSGQVEYITGPAVTTYRAVVKYRCNEFYTMTTNDGK + G+

Sbjct 363 IVDCGPPDDLPSGQVEYITGPAVTTYRAVVKYRCNEFYTMTTNDGKYVCEADGF 416

-----------------------------------------------------------------------------------------

genewise $Name: wise2-2-0 $ (unreleased release)

This program is freely distributed under a GPL. See source directory

Copyright (c) GRL limited: portions of the code are from separate copyright

Query protein: Q2KJ45_BOVIN

Comp Matrix: blosum62.bla

Gap open: 12

Gap extension: 2

Start/End local

Target Sequence Bt.22320.2.S1_at

Strand: forward

Start/End (protein) local

Gene Paras: human.gf

Codon Table: codon.table

Subs error: 1e-05

Indel error: 1e-05

Model splice? model

Model codon bias? flat

Model intron bias? tied

Null model syn

Algorithm 623

genewise output

Score 11.11 bits over entire alignment

Scores as bits over a synchronous coding model

Warning: The bits scores is not probablistically correct for single seqs

See WWW help for more info

Q2KJ45_BOVIN 224 KPFR

KPFR

KPFR

Bt.22320.2.S1_a 39 acta

actg

atta

//

Gene 1

Gene 39 50

Exon 39 50 phase 0

//

>Bt.22320.2.S1_at.[39:50].sp.tr

KPFR

//
