# Supplementary material for: AIGO: Towards a unified framework for the Analysis and the Inter-comparison of GO functional annotations
Source: BMC Bioinformatics. 2011 Nov 3;12:431. doi: 10.1186/1471-2105-12-431 (PMC3237112; doi:10.1186/1471-2105-12-431)
Supplement: Additional file 4 — Rice protein with suspicious annotations. [file 1471-2105-12-431-S4.DOC]

**Rice protein with suspicious annotations**

We have identified only one protein, named Igag_0167 (UniProt accession [E0SPZ6](http://www.uniprot.org/uniprot/E0SPZ6)), in the GOA database being annotated to the two GO terms GO:0003735 (structural constituent of ribosome) and GO:0004629 (phospholipase C activity). It is likely that the annotation to GO:0004629 is an error due to a match to a protein domain: IPR000909 Phospholipase C, phosphatidylinositol-specific, X domain.

The SQL query used to identify the protein Igag_0167 was:

SELECT
gene_product.symbol AS gp_symbol,
gene_product.symbol AS gp_full_name,
dbxref.xref_dbname AS gp_dbname,
dbxref.xref_key AS gp_acc,
species.genus,
species.species,
species.common_name
FROM term
INNER JOIN graph_path ON (term.id=graph_path.term1_id)
INNER JOIN association ON (graph_path.term2_id=association.term_id)
INNER JOIN gene_product ON (association.gene_product_id=gene_product.id)
INNER JOIN species ON (gene_product.species_id=species.id)
INNER JOIN dbxref ON (gene_product.dbxref_id=dbxref.id)
WHERE
term.name = 'phospholipase C activity'
AND
is_not=0
AND
EXISTS
 (SELECT *
  FROM association AS a2
  INNER JOIN graph_path AS tc2 ON (tc2.term2_id=a2.term_id)
  INNER JOIN term AS t2 ON (tc2.term1_id=t2.id)
  WHERE a2.gene_product_id = association.gene_product_id
  AND   t2.name = 'structural constituent of ribosome'
  AND   a2.is_not=0)
