# Supplementary material for: AIGO: Towards a unified framework for the Analysis and the Inter-comparison of GO functional annotations
Source: BMC Bioinformatics. 2011 Nov 3;12:431. doi: 10.1186/1471-2105-12-431 (PMC3237112; doi:10.1186/1471-2105-12-431)
Supplement: Additional file 5 — Annotations of Os.2243.1.S1_a_at. [file 1471-2105-12-431-S5.DOC]

**Annotations of Os.2243.1.S1_a_at**

In revision 20 of the Affymetrix annotations for the rice array, the annotation file for the probe-set Os.2243.1.S1_a_at contains:

...

Alignments chr05|11682:1504392-1510196 (+) // 99.67 //

SwissProt Q9ZST0

...

In revision 31, the annotation file for the probe-set Os.2243.1.S1_a_at contains:

...

Alignments chr5:1535747-1541551 (+) // 99.67 //

SwissProt Q75IL8

...

After verification in UniProt, the GO annotations of release 20 correspond to the annotations of the 50S ribosomal protein RPL5 (Q9ZST0), while the GO annotations of release 31 correspond to the annotations of the phospholipase C P0683F12.11 (Q75IL8). To identify which of these two annotations is wrong, we use Genewise (Birney *et al.*, 2004)to compare the nucleotide sequence given by Affymetrix for the probe-set Os.2243.1.S1_a_at to the sequences of the two proteins mentioned above.

The results, showing that the information in release 31 is correct, are:

genewise $Name: wise2-2-0 $ (unreleased release)

This program is freely distributed under a GPL. See source directory

Copyright (c) GRL limited: portions of the code are from separate copyright

Query protein: RK5_ORYSJ

Comp Matrix: blosum62.bla

Gap open: 12

Gap extension: 2

Start/End local

Target Sequence Os.2243.1.S1_a_at;

Strand: forward

Start/End (protein) local

Gene Paras: human.gf

Codon Table: codon.table

Subs error: 1e-05

Indel error: 1e-05

Model splice? model

Model codon bias? flat

Model intron bias? tied

Null model syn

Algorithm 623

genewise output

Score 16.10 bits over entire alignment

Scores as bits over a synchronous coding model

Warning: The bits scores is not probablistically correct for single seqs

See WWW help for more info

RK5_ORYSJ 37 RRALRVAASAAADAPPKPAPPPTSPS

R A R +S A AP A SPS

RPACRCGSSGEASAPSGSATTAASPS

Os.2243.1.S1_a_ 128 acgtctgatgggtgctgtgaaggact

gccgggggcgacccccgccccccgcc

atccgtgcccgactgactgccggccc

//

Gene 1

Gene 128 205

Exon 128 205 phase 0

//

>Os.2243.1.S1_a_at;.[128:205].sp.tr

RPACRCGSSGEASAPSGSATTAASPS

//

-----------------------------------------------------------------------------------------

This program is freely distributed under a GPL. See source directory

Copyright (c) GRL limited: portions of the code are from separate copyright

Query protein: Q75IL8_ORYSJ

Comp Matrix: blosum62.bla

Gap open: 12

Gap extension: 2

Start/End local

Target Sequence Os.2243.1.S1_a_at;

Strand: forward

Start/End (protein) local

Gene Paras: human.gf

Codon Table: codon.table

Subs error: 1e-05

Indel error: 1e-05

Model splice? model

Model codon bias? flat

Model intron bias? tied

Null model syn

Algorithm 623

genewise output

Score 210.46 bits over entire alignment

Scores as bits over a synchronous coding model

Warning: The bits scores is not probablistically correct for single seqs

See WWW help for more info

Q75IL8_ORYSJ 519 KMDTWIPAWDHEFEFPLSVPELALLRVEVHESDNHQKDDFGGQTCLPVW

KMDTWIPAWDHEFEFPLSVPELALLRVEVHESDNHQKDDFGGQTCLPVW

KMDTWIPAWDHEFEFPLSVPELALLRVEVHESDNHQKDDFGGQTCLPVW

Os.2243.1.S1_a_ 1 aagatacgtgcgtgtctagcgcgcccgggcgtgaccaggtggcatccgt

atacgtccgaaatatctgtcatcttgtataacaaaaaaatggacgtctg

ggcggtgagtcgcgcggcggggggcggggcgccccggccccggccgggg

Q75IL8_ORYSJ 568 ELRRGIRSVRLCDHRGEPLRSVKLLMRFDFT

ELRRGIRSVRLCDHRGEPLRSVKLLMRFDFT

ELRRGIRSVRLCDHRGEPLRSVKLLMRFDFT

Os.2243.1.S1_a_ 148 gcccgactgactgccggccctgaccactgta

atgggtgctgtgaaggactgctatttgtatc

gcgacccccgccccccgcccccgccgccccc

//

Gene 1

Gene 1 240

Exon 1 240 phase 0

//

>Os.2243.1.S1_a_at;.[1:240].sp.tr

KMDTWIPAWDHEFEFPLSVPELALLRVEVHESDNHQKDDFGGQTCLPVWELRRGIRSVRL

CDHRGEPLRSVKLLMRFDFT

//
